# Unusual Appearance of Meyerson Phenomenon Arising From a Congenital Melanocytic Nevus: Case Report and Literature Review

**DOI:** 10.7759/cureus.68021

**Published:** 2024-08-28

**Authors:** Khulood Abdulraouf Almarzooqi, Shaden Abdelhadi

**Affiliations:** 1 Department of Dermatology, Sheikh Khalifa Medical City, Abu Dhabi, ARE

**Keywords:** atopic dermatitis, weeping eczema, congenital melanocytic nevus, halo nevus, halo dermatitis, meyerson nevus

## Abstract

Although melanocytic nevi have a relatively uneventful course throughout their existence, some may develop an inflammatory reaction known as the Meyerson phenomenon. Initially, the Meyerson phenomenon has been exclusively described in melanocytic nevi. However, it has since been observed in both pigmented and non-pigmented lesions, thus expanding the description from Meyerson nevus to the phenomenon. We report a case of an unusual Meyerson phenomenon arising from a congenital melanocytic nevus characterized by a weeping eczematous response.

## Introduction

Meyerson phenomenon, also known as halo dermatitis or halo eczema, describes the development of an eczematous reaction around a central cutaneous lesion [1]. Since its first description by Meyerson in 1971, many cases with predominantly benign dermatological entities have been reported, with melanocytic lesions being more common than non-melanocytic ones. Classically, it presents as a single, pruritic, symmetrical, scaly erythematous halo surrounding a pre-existing pigmented nevus on the trunk and proximal extremities [2]. Histology often shows focal parakeratosis, irregular acanthosis, spongiosis, and a perivascular lymphocytic infiltrate with eosinophils [2]. We present an unusual case of Meyerson's phenomenon, characterized by a weeping eczematous response.

## Case presentation

**Figure 1:**
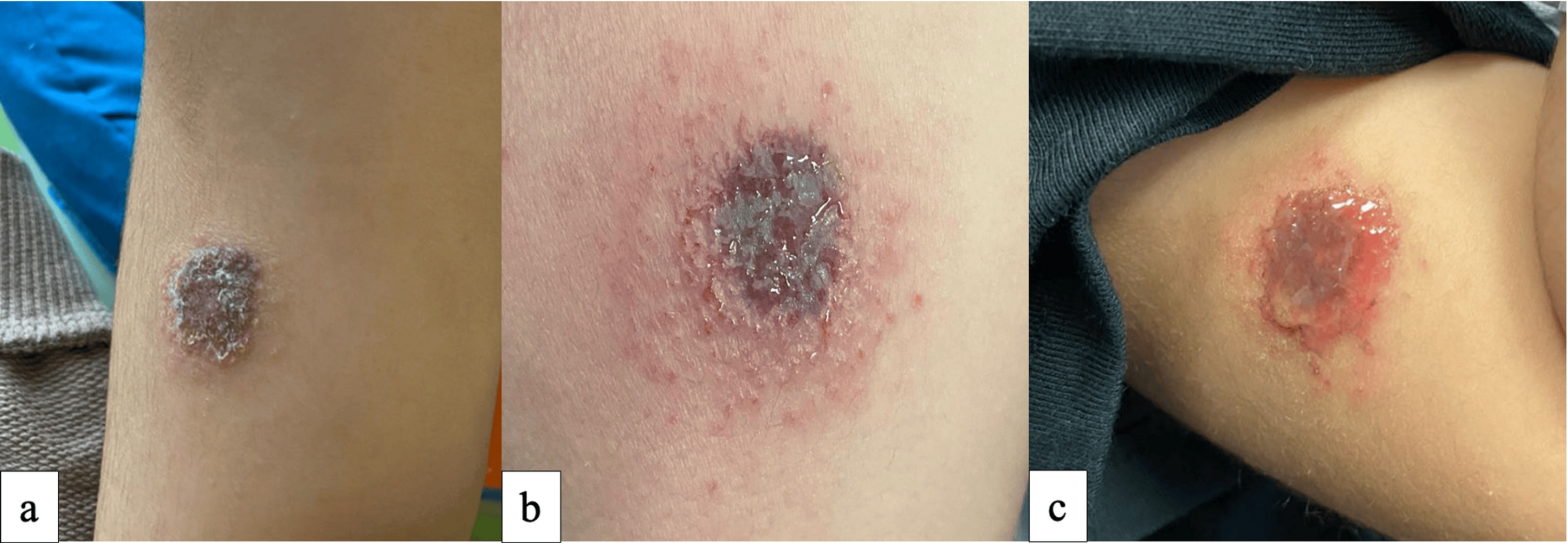
Clinical images showing the progression of the melanocytic lesion, which presents as a 2.0 × 2.5 cm dark brown plaque (a), with surrounding honey-crusted erythematous plaque (b) and serous discharge (c).

A 6-month-old male presented with a 2-month history of a pruritic rash overlying and surrounding a pigmented skin lesion on the right upper arm, associated with clear watery discharge and erythema, as seen in Fig. 1. No bleeding from the lesion was noted. The pigmented skin lesion was present since birth as a dark brown, slightly raised plaque with well-circumscribed borders and no hypertrichosis. The lesion’s growth had been proportional to the growth of the limb before the presentation. He is known to have atopic dermatitis. Otherwise, his mother denied any trauma, insect bite, or vaccination on or near that site. On physical examination, a 2.0 × 2.5 cm dark brown plaque with watery serous discharge and a surrounding rim of erythematous honey-crusted plaque. Additionally, a mildly active eczematous patch on the chin was also seen. An initial impression of halo eczema and atypical nevus was made. Still, other possibilities, such as solitary mastocytoma, were considered, especially because the primary nevus was not very clear due to the severity of the eczematous reaction. Therefore, a skin biopsy for hematoxylin and eosin, as well as a swab for bacterial culture, were obtained. 

**Figure 2:**
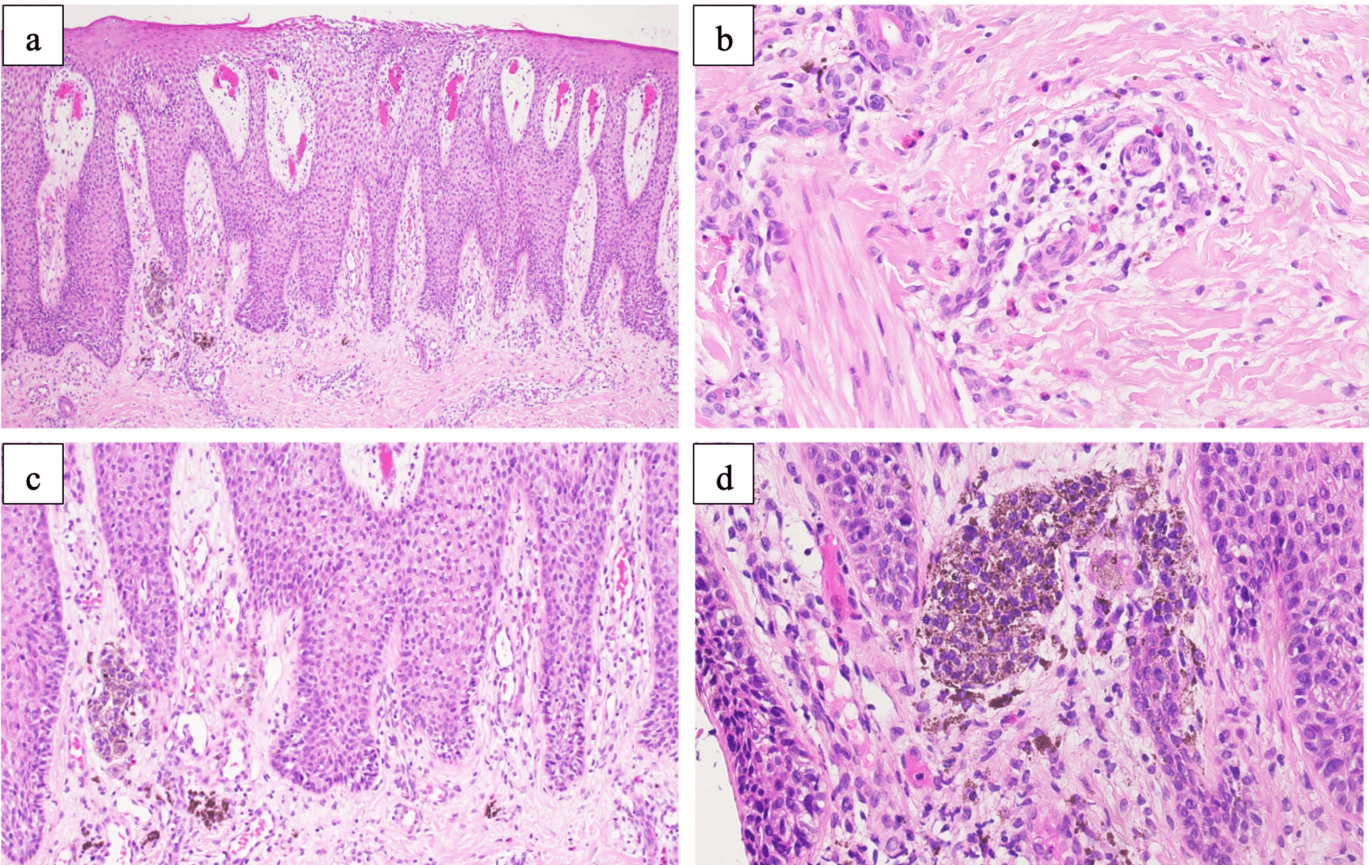
Histologic sections showing psoriasiform epidermal hyperplasia with mild spongiosis (a), eosinophils infiltrate (b), Junctional nests of nonatypical melanocytes (c, d)

Histological examination revealed psoriasiform epidermal hyperplasia, as shown in Fig. 2. The epidermis displayed spongiosis and exocytosis of lymphocytes, some polymorphonuclear cells, and occasional eosinophils. Furthermore, there was a lympho-eosinophilic infiltrate around the vessels, with scattered eosinophils noted within the deeper dermis. Mast cells appeared increased on CD117 stain. However, they did not form dense aggregates to suggest solitary mastocytoma. This increased number is interpreted as hyperplastic in nature, as may occur in inflammatory dermatoses, including spongiotic dermatitis. Junctional nests of melanocytes are also appreciated with no atypia. The skin swab culture showed moderate growth of *Staphylococcus aureus*.

**Figure 3:**
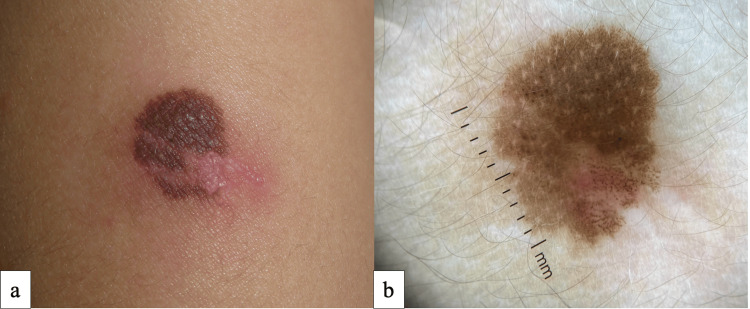
Post-biopsy clinical (a) and dermoscopic images (b) at follow-up examination, the congenital melanocytic nevus can be appreciated after 2 weeks of treatment with a topical corticosteroid.

Treatment was initiated with a topical combination of fusidic acid 2% and betamethasone 0.05%. At a 2-week follow-up visit, impetiginized eczema and serous discharge had resolved, as seen in Fig. 3. Dermoscopic examination revealed a diffuse homogeneous brown pigment network with few smaller central brown globules, perifollicular hypopigmentation, and scarring, as shown in Fig3.

## Discussion

While the Meyerson phenomenon has previously been observed in adults with acquired melanocytic nevi, there have been more recent reports of the same clinical presentation in children with congenital nevi [1]. It should be noted that it is also not exclusive to benign melanocytic nevi. It can also occur in atypical nevi and melanoma [3, 4]. It’s been reported with various non-melanocytic lesions, including seborrheic keratoses, vascular malformations, molluscum contagiosum, nevus sebaceous, dermatofibromas, stucco keratosis, lentigo, keloid, and insect bites, as well as basal cell and squamous cell carcinoma [1]. 

Although the exact pathophysiology of this phenomenon is unknown, experts believe this is an immune-mediated process triggered by factors such as ultraviolet exposure, interferon treatment, or viral infections [1]. The proposed hypothesis for the former is that UV exposure may have altered the expression of antigenic sites on nevus cells, subsequently leading to an immune reaction against them [2]. While cases reported following interferon treatment or COVID-19 infection were attributed to the upregulation of specific adhesion molecules such as the intercellular cell adhesion molecule 1 (ICAM-1) and their subsequent interaction with CD4 T-cells [5]. Further, possible Koebner reactions in the form of the Meyerson phenomenon have been reported after using pulsed dye lasers for the treatment of port-wine stains (PWS). The vasodilatation and ectasia associated with PWS, as well as the trauma from the laser, lead to an increase in proinflammatory mediators, which can either unmask an underlying atopic diathesis or exacerbate existing eczematous lesions [6].

The diagnosis can be clinically established by observing a pruritic and erythematous scaly halo that encircles a pre-existing melanocytic nevus or other lesions. The change may involve one or more nevi simultaneously and usually appears on the trunk and proximal extremities [1, 2]. It is more prevalent in young adults with an even gender distribution [1]. The link between Meyerson nevus and atopic dermatitis is not well-established. However, as seen in our case and other case reports, the presence of eczematous lesions in other locations in patients with atopic backgrounds has been observed [1]. 

Meyerson nevus or halo dermatitis should not be confused with halo nevus. Unlike halo nevus, the original nevus appears unchanged following the resolution of the dermatitis [7]. However, the evolution of a Meyerson nevus into a halo nevus has been reported [8], as well as the coexistence of both types of nevi in the same patient [9]. 

Histopathologically, it is characterized by a subacute spongiotic inflammatory reaction with variable acanthosis, parakeratosis, and a dermal inflammatory infiltrate composed of mononuclear cells and eosinophils [2]. The inflammatory cell infiltrate consists predominantly of CD4+ over CD8+ T lymphocytes, suggesting a possible immune-mediated reaction [2]. Compared to halo nevus, there is no histologic evidence of regression. Of note, halo nevus often shows a dense inflammatory infiltrate mainly by CD8+ T lymphocytes [2]. 

Topical corticosteroids are the most common treatment options for intense inflammation and itch. Observation is also reasonable, as most cases resolve spontaneously within a few weeks without change to the central nevus [1]. Nonetheless, in cases that do not respond to corticosteroid treatment, excision of the central lesion has been shown to resolve the eczema [10]. As for long-term follow-up, recurrence of inflammation has been commonly reported within the same lesion and/or other lesions [7].

## Conclusions

In summary, descriptions of this entity are rare, even more so in pediatrics, and its frequency is probably underestimated. The exact relationship between Meyerson nevus and atopic dermatitis is not well established, and more research is needed to determine whether the two conditions are associated. Finally, healthcare professionals must be aware that watery serous discharge or secondary impetiginization may be the presenting clinical signs, in addition to classic active eczema. 
